# Trends in amount of use to upper limb function in patients with subacute stroke: a cross-sectional study using segmental regression analysis

**DOI:** 10.1186/s12883-023-03469-z

**Published:** 2023-12-04

**Authors:** Koichiro Hirayama, Marina Matsuda, Moe Teruya, Takeshi Fuchigami, Shu Morioka

**Affiliations:** 1 Department of Rehabilitation, Eishinkai Kishiwada Rehabilitation Hospital, 8-10, Kanmatsucho, Kishiwada, 596-0827 Osaka Japan; 2 Graduate School of Comprehensive Rehabilitation, Osaka Prefecture University, Osaka, Japan; 3 Stroke Rehabilitation Research Laboratory, Eishinkai Kishiwada Rehabilitation Hospital, Osaka, Japan; 4 Neurorehabilitation Research Center, Kio University, 4-2-2 Umaminaka, Koryo, Kitakatsuragi-gun, Nara, 635-0832 Japan

**Keywords:** Stroke, Paretic upper limb, Segmental regression analysis

## Abstract

**Background:**

Hemiparesis affects approximately 33–80% of patients with stroke, and a quarter of these individuals experience difficulty with the voluntary use of their paretic upper limb for performing activities of daily living within five years of stroke onset. Therefore, assessing upper limb functionality and use after a stroke is crucial. The Fugl-Meyer Assessment (FMA) and the Motor Activity Log (MAL) are the two most widely used methods for assessing post-stroke paretic upper limb. While previous research has shown a strong correlation between the FMA of Upper Extremity (FMA-UE) and the MAL scores, to date, no study has investigated the differences in the characteristics and trends of upper extremity usage frequency in the FMA-UE. This study aimed to statistically categorize the FMA-UE scores using segmental regression analysis and identify disparities in the trends of paretic upper extremity utilization frequency in MAL.

**Methods:**

Patients with first-episode subacute stroke were chosen for the cohort study. The primary assessments used were FMA-UE and MAL Amount of Use (MAL-A); age, gender, and time since onset served as secondary assessments. Segmental regression analysis was used, with FMA-UE as the independent variable and MAL-A as the dependent variable. R^2^ values were calculated using linear and polynomial regression on binary values, and the coefficients of determination were compared using segmental regression analysis.

**Results:**

The study included 203 participants with a mean age of 70.1 ± 13.1 years; 113 were male and 90 female. The mean time since onset was 29.2 ± 14.8 days, the mean FMA-UE score was 43.6 ± 22.3 points, and the mean MAL-A score was 2.3 ± 2.0 points. The segmental regression analysis revealed that the inflection point for FMA-UE was 45.3 points, and the slope of the regression line underwent a transformation before and after the inflection point.

**Conclusions:**

This study indicates that the trend in the amount of use of paretic upper limb utilization changes around inflection point 45 in the FMA-UE. These findings could be useful for designing rehabilitation strategies to improve paretic upper limb utilization by increasing exercise duration in patients with subacute stroke.

## Background

Recent findings indicate that 33–80% of patients with stroke experience upper limb dysfunction due to paretic within 6 months following a stroke [1, 2]. This condition is associated with the quality of life of patients and disrupts their activities of daily living (ADLs), emphasizing the need for appropriate rehabilitation [3, 4]. A study revealed that approximately 80% of patients with stroke can expect to regain the function in their paretic upper limb within three weeks of stroke onset [5]. However, consistent non-use of the paretic upper limb in ADLs may worsen their functional decline [6]. Thus, monitoring the usage of the paretic upper limb, as can help assess any decline in functionality.

The Fugl-Meyer Assessment of Upper Extremity (FMA-UE) is a widely recognized tool for evaluating upper extremity function [7], while the Motor Activity Log 14 (MAL) is commonly used to assess usage behavior in paretic upper extremities [8]. Both assessments have demonstrated high reliability and validity [9, 10].

Schweighofer et al. performed a comprehensive reanalysis of data from individuals participating in a large randomized controlled trial to assess the effectiveness of constraint-induced movement therapy, validating a computer simulation-based recovery model for paretic upper limb. This study showed that the Wolf Motor Function Test (WMFT), scored one week after treatment, effectively predicted the use of the paretic upper limb one year post-treatment, and surpassing a specific WMFT score indicated significantly increased use of the limb [11]. These results imply that the increased frequency of paretic upper limb use in ADLs may be accompanied by an increase in the duration of use and that a certain level of upper limb function is necessary to elevate the frequency of paretic upper limb use in ADLs. However, this study focused specifically on patients with stroke treated for paretic upper limb and may not be generalizable to all patients with stroke in clinical practice.

To develop effective rehabilitation strategies that can enhance the duration and amount of use of the paretic upper limb use in patients with stroke, to evaluate using the widely accepted FMA-UE and MAL amount of use (MAL-A) variables in clinical practice.

We hypothesized that segmental regression analysis, a statistical tool adept at uncovering alterations and trends in the relationship between two variables, could be employed to understand the connection between FMA-UE and MAL-A. This analytical approach is particularly effective when independent variables are categorized into diverse groups, thus revealing various relationships among variables within each category [12]. Consequently, using segmental regression analysis, this study aimed to elucidate the correlation between the amount of use of the paretic upper limb and its function in patients with subacute stroke.

## Methods

The aim of this cross-sectional study was to identify prevailing patterns of MAL-A, as evaluated using the FMA-UE, which is a functional assessment tool used to evaluate patients coping with subacute stroke-related paretic upper extremity. This study is reported following the STROBE guidelines [ref: https://www.strobe-statement.org/].

### Participants

Individuals who had suffered an ischemic or hemorrhagic stroke were eligible for participation in this study. The study was conducted between April 2019 and March 2022 at the Kishiwada Rehabilitation Hospital in Osaka, Japan. Inclusion criteria were: at least 18 years of age and within 3 months of their first stroke. The exclusion criteria were: having severe impairments in communication or memory, which would hinder adherence to evaluation protocols.

Participants underwent a rehabilitation program consisting of physical therapy, occupational therapy, and speech-language pathology therapy. Each session lasted between 40 and 80 min, with a total duration of 2–3 h per day, daily. The treatment program included fundamental movement exercises, such as standing and sitting, balance training, activities of self-care, such as toileting, dressing, bathing, upper limb function training, and increased brain function exercises. Occupational therapy, primarily responsible for upper limb function training, involves manual therapy for conditioning the upper limbs using automatic assistance exercises and task-specific exercises.

### Outcome measures

The FMA-UE consists of four sub-items with a total of 33 items that are rated on a 3-point ordinal scale (0: impossible, 1: partially possible, and 2: possible) [7]. The reliability and validity of the FMA-UE have been studied extensively and high reliability and content validity have been reported [7, 13]. The amount of use of the paretic upper limb in ADLs was assessed using the MAL-A, which consists of 14 items rated on a 5-point ordinal scale (from 0: no use at all to 5: usage same as before injury). The MAL was developed by Taub et al., and the Japanese version reported by Takahashi et al. was used in this study [4, 14]. Occupational therapists supervised and evaluated the participants within a week of admission, and data, including hemisphere and site of injury, and time since onset were obtained from their medical records.

### Statistical analyses

For statistical analysis, descriptive statistics, including n-number, mean, and standard deviation (SD) were calculated for basic information, such as age, sex, hemisphere of injury, type of stroke, and number of days since onset. The normality of the two variables was assessed using the Shapiro–Wilk test, and a Pearson or Spearman correlation analysis was performed depending on normality.

Segmented regression analysis is a statistical technique used when independent variables categorized into distinct groups exhibit varied relationships among the variables in these categories. The interval between independent variable categories is calculated as an inflection point. An inflection point, in segmented regression analysis, is a specific point that may indicate a change from a previously established pattern. The fitment of break points in segmented regression analysis was evaluated using the Akaike information criterion (AIC), a measure of a model’s fitness, where a smaller AIC value indicates a better fit to the ideal model [15].

In this study, a scatter plot was generated based on the FMA-UE and MAL-A variables, and segmented regression analysis was performed, with FMA-UE as the independent variable and MAL-A as the dependent variable. The breakpoint(s) in FMA-UE were calculated from the results of the analysis. AIC values were calculated for models with a variable number of breakpoints to determine the optimal number of breakpoint(s) to best model the collected data. Additionally, R^2^ values were computed based on the linear and polynomial regression models to compare the fit with other regression analyses, and the coefficients of determination were compared. The statistical software used was R ver. 4.1.1 software, *p* < 0.05 was considered statistically significant.

## Results

**Fig. 1:**
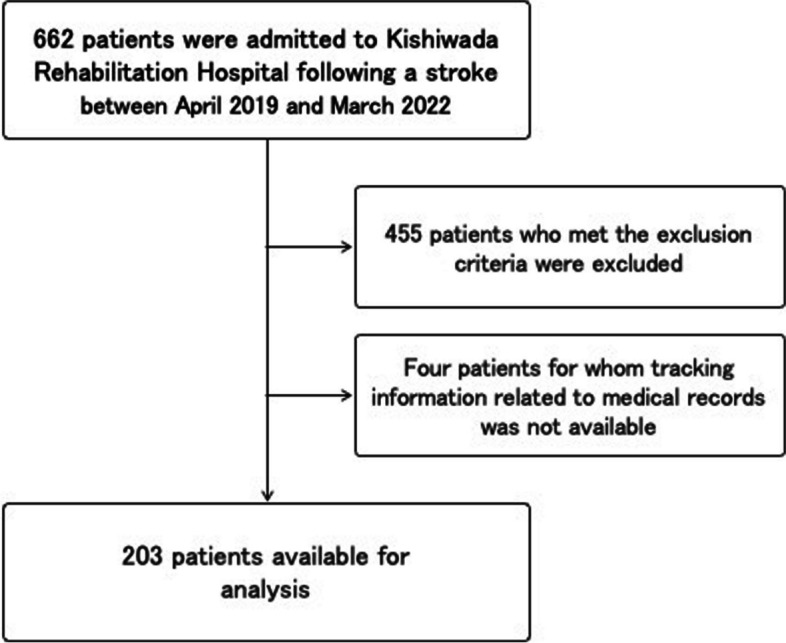
Patient exclusion flowchart

Out of the 662 patients with stroke admitted to Kishiwada Rehabilitation Hospital between April 2019 and March 2022, 203 patients were selected for analysis after carefully considering the inclusion and exclusion criteria (Fig. 1). The characteristics of the study participants are detailed in Table 1. Their mean age was 70.1 years (SD 13.1; range 19–94 years), with an average time of 29.2 days (SD 14.8 range 8–99 days) since stroke onset. The time from admission to evaluation averaged 4.3 days with a standard deviation of 9.0 days. No outliers were observed in FMA-UE and MAL-A.

**Table 1:** Characteristics of the patients in this study

Characteristic (*n* = 203)	n, mean ± standard deviation
Age (years)	70.1 ± 13.1
Man/woman	113 / 90
Hemisphere	
Right	89
Left	114
Type of stroke	
Hemorrhagic	50
Ischemic	153
Time since stroke onset(days)	29.2 ± 14.8
FMA-UE(points)	43.6 ± 22.3
MAL-A(points)	2.3 ± 2.0
MMSE(points)	24.4 ± 4.8

*FMA-UE* Fugl-Meyer Assessment, *MAL-A* Motor Activity Log Amount of Use, *MMSE* Mini-Mental State Examination

**Fig. 2:**
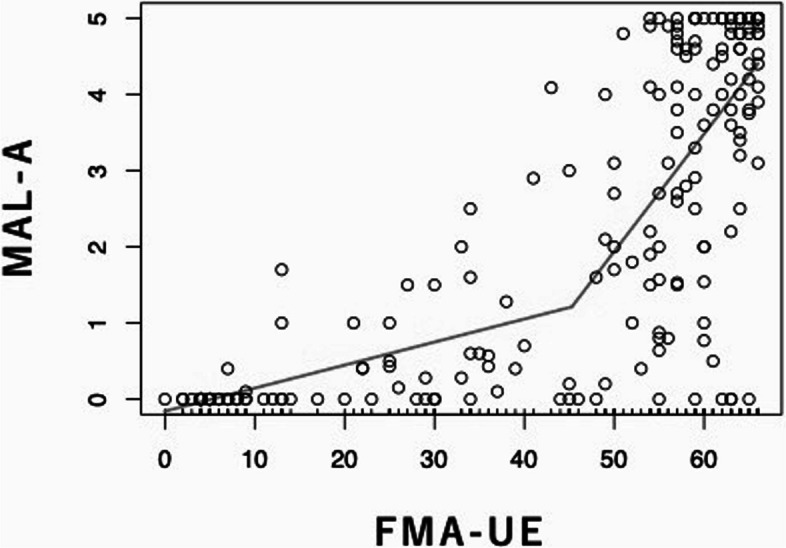
Segment regression analysis based on FMA-UE and MAL-A plots. The gradient of the regression line was x = 0.03 below the point of inflection and x = 0.12 above the point of inflection. The coefficient of determination was 0.65

The Shapiro–Wilk test indicated non-normality in FMA-UE (*p* < 0.01) and MAL-A (*p* < 0.01), thus Spearman’s correlation analysis was used. The resulting positive correlation coefficient was 0.79 (*p* < 0.01), which was employed to perform a segmented regression analysis by plotting the FMA-UE and MAL-A values (Fig. 2).

The inflection point for FMA-UE was 45.3, and the slope of the regression line for MAL-A increased after 45.3 for FMA-UE compared to that for MAL-A below 45.3. AIC values for the number of breakpoints revealed the most accurate model should contain one inflection point (AIC values: 660.6) even when three or more breakpoints were present. The coefficients of determination for segment, linear, and polynomial regression analyses were 0.65, 0.59, and 0.64, respectively. The linear regression model was computed as y = 0.0696x − 0.7828, while the polynomial regression model was calculated as y = 0.0015 × ^2^ − 0.0348x + 0.2248.

## Discussion

The most important finding of this study is that an FMA-UE score of 45.3 may indicate a shift in the pattern of usage of the paretic upper limb in ADLs, which was assessed using the MAL-A. This critical score was derived from a segmental regression analysis of a cross-sectional evaluation of functionality and amount of use of the affected upper limb in patients with subacute stroke.

Previous studies have shown that the amount of use of the paretic upper extremity in ADLs is significantly impacted by the stage of recovery of the patient [8, 14, 16]. Our study findings also support this observation, and the findings indicate a strong correlation (correlation coefficient 0.79) between FMA-UE and MAL-A. By analyzing the two segments, we found that patients with stroke who scored 45.3 or higher on the FMA-UE tended to use their paretic upper limb more frequently than those who scored lower than 45.3. This suggests that the amount of use is affected by the level of upper limb function and that changes in the amount of use can be detected using the FMA-UE score.

This study performed a segmented regression analysis with FMA-UE as the independent variable and MAL-A as the dependent variable, with the assumption of linearity of the regression analysis. The breakpoint, which is the point at which the sum of the residuals’ squares is minimized by fitting a regression line to each segment, was identified. The fitting of the segment regression line was determined by the number of breakpoints, and AIC was used to evaluate the fitting. The results showed that the regression line with a single break point was ideal. Moreover, the coefficient of determination for polynomial regression analysis was 0.64, which was not significantly different from the coefficient of determination for segmented regression analysis (0.65). This suggests that the latter is valid and that the trend of the amount of use of the paretic upper limb in MAL-A may change after the breakpoint.

Although the relationship between the function and amount of use of the paretic upper limb is believed to be closely associated, recent studies suggest that the correlation between the two may not be linear; a minimum threshold of upper extremity function might be necessary for long-term use [11, 17]. Chin et al. categorized patients with stroke based on the severity of paretic upper extremity scored using the FMA-UE (mild: 51–66, moderate: 23–50, and severe: 0–22). Accelerometers were used to examine upper limb usage patterns of participants with varying levels of functional deficiencies after placing them in their respective groups based on the FMA-UE score. The study revealed that 63% of participants with mild impairment, 13.3% with moderate impairment, and none with severe impairment used their paretic upper limb for more than 6 h per day. However, the amount of use varied characteristically in each case [18]. These findings indicate the presence of a threshold point in FMA-UE, where the pattern of use changes with different functional levels. The inflection point identified in our study may represent the point of divergence at which the pattern of use changes.

To the best of our knowledge, this the first study to use the FMA-UE and MAL-A, widely used in clinical practice, to determine the functioning and use patterns of the paretic upper limb in patients with subacute stroke.

The inflection point determined for FMA-UE in this study can serve as a guideline for shifting the focus from functional skills intervention to activity and participation based intervention for paretic upper limb, as the point reflects an inclination towards more frequent use of the paretic side of the upper limb. Additionally, the primary endpoints, namely FMA-UE and MAL, which are used more frequently than other upper extremity functional assessments [19], can significantly impact clinical evaluation and intervention.

This study has four limitations. Firstly, the regression analysis was performed based only on two variables, FMA-UE and MAL-A; both of which may have confounding factors. Secondly, the data was sampled exclusively from a single center, which may not be an accurate representation of the general population for which this study was intended. Third, hand function, which affects the amount of use in the paretic upper extremity, was not tracked as a subitem of the FMA-UE in this study. Fourth, in segmented regression analysis, it is common to treat the data being applied as continuous variables. While some studies treat assessments like FMA-UE or MAL-A as interval scales, the results of this study, which adapted them as ordinal scales, must be interpreted with caution. Consequently, future verification of the data by sampling at multiple centers and employing a large sample size, and FMA-UE sub-items is imperative.

## Conclusions

Using two variables, FMA-UE and MAL-A, we performed a segmented regression analysis. Our findings suggest that the trend of amount of use of the paretic upper extremity may change after reaching 45.3 points on the FMA-UE. This novel finding could potentially aid in the development of rehabilitation strategies aimed at enhancing paretic upper limb utilization by increasing the exercise duration in subacute stroke patients.

## Data Availability

The manuscript includes a comprehensive description of all pertinent data for this study. The data is securely coded and protected by a PIN code accessible solely by the principal investigator. The Research Ethics Committee of Kishiwada Rehabilitation Hospital forbids the involvement of any third party in the handling of the study’s data. In the event of an inquiry, Kouichiro Hirayama, the corresponding author, is available to provide further insight into the statistical computations carried out during this study.

## Abbreviations

ADLs: Activities of daily living
FMA: Fugl-Meyer Assessment
FMA-UE: Fugl-Meyer Assessment of Upper Extremity
MAL: Motor Activity Log
MAL-A: Motor Activity Log amount of use
WMFT: Wolf Motor Function Test
SD: Standard Deviation
AIC: Akaike information criterion
MMSE: Mini Mental Standard Exam
